# Suicide Risk Evaluations and Suicide in the Veterans Health Administration

**DOI:** 10.1001/jamanetworkopen.2024.61559

**Published:** 2025-02-25

**Authors:** Kevin G. Saulnier, Courtney L. Bagge, Dara Ganoczy, Nazanin H. Bahraini, Jennifer Jagusch, Avinash Hosanagar, Mark A. Ilgen, Paul N. Pfeiffer

**Affiliations:** 1 Veterans Affairs (VA) Serious Mental Illness Treatment Resource and Evaluation Center, Office of Mental Health, Ann Arbor, Michigan; 2 Department of Psychiatry, University of Michigan, Ann Arbor; 3 VA Center for Clinical Management Research, Health Systems Research, Ann Arbor, Michigan; 4 VA Rocky Mountain Mental Illness Research, Education, and Clinical Center for Suicide Prevention, Aurora, Colorado; 5 Department of Physical Medicine and Rehabilitation, Anschutz Medical Campus, University of Colorado, Aurora; 6 Department of Psychiatry, Anschutz Medical Campus, University of Colorado, Aurora; 7 Ann Arbor VA Healthcare System, Ann Arbor, Michigan

## Abstract

**Importance:**

The Veterans Health Administration (VHA) implemented the Comprehensive Suicide Risk Evaluation (CSRE) in 2019 to standardize suicide risk assessment across the health care system. Identifying CSRE responses associated with suicide could inform risk management and prevent suicide.

**Objective:**

To identify CSRE responses associated with subsequent suicide.

**Design, Setting, and Participants:**

This cohort study examines acute (within 30 days) and chronic (within 365 days) suicide risk after 269 374 CSREs were administered. Participants included US VHA patients undergoing CSRE evaluation between November 1, 2019, and December 31, 2020. Data collection and analysis were performed from April 5 to August 20, 2024.

**Exposures:**

CSRE responses, including suicidal ideation, behaviors, warning signs, risk factors, and protective factors.

**Main Outcome and Measure:**

Suicide per death certificate data from the Department of Veterans Affairs and Department of Defense Mortality Data Repository. Outcomes were analyzed using multivariable Cox proportional hazards regression.

**Results:**

A total of 153 736 patients with 269 374 valid CSREs (86.26% male; mean [SD] age, 50.48 [15.26] years) were included in the multivariable-adjusted analyses of suicide. Suicidal ideation (hazard ratio [HR], 3.14; 95% CI, 1.51-6.54), firearm access (HR, 2.62; 95% CI, 1.49-4.61), making preparations for a suicide attempt (HR, 2.15; 95% CI, 1.27-3.62), seeking access to lethal means (HR, 2.04; 95% CI, 1.11-3.75), anxiety (HR, 1.80; 95% CI, 1.16-2.81), and psychiatric hospitalization history (HR, 1.63; 95% CI, 1.02-2.61) were associated with increased suicide risk within 30 days, whereas anger (HR, 0.50; 95% CI, 0.30-0.85) was associated with decreased risk. Suicidal ideation (HR, 1.63; 95% CI, 1.20-2.21), firearm access (HR, 1.55; 95% CI, 1.13-2.13), making preparations for a suicide attempt (HR, 1.56; 95% CI, 1.09-2.23), reckless behaviors (HR, 1.40; 95% CI, 1.00-1.95), and history of psychiatric hospitalization (HR, 1.68; 95% CI, 1.32-2.13) were associated with increased suicide risk within 365 days, whereas anger (HR, 0.56; 95% CI, 0.44-0.71), preexisting risk factors (eg, trauma; HR, 0.77; 95% CI, 0.62-0.96), and recent transition from the military (HR, 0.39; 95% CI, 0.22-0.70) were associated with decreased risk in multivariable-adjusted analyses. Suicide risk within 365 days was elevated among patients identified by clinicians to be at intermediate acute risk (HR, 1.39; 95% CI, 1.13-1.71), intermediate chronic risk (HR, 1.34; 95% CI, 1.01-1.77), and high chronic risk (HR, 1.74; 95% CI, 1.22-2.47) of suicide compared with patients at low risk in multivariable-adjusted analyses.

**Conclusions and Relevance:**

In this cohort study of suicide following risk assessments, findings suggest that only a few constructs are associated with subsequent suicide, particularly suicidal ideation, firearm access, and preparatory behaviors. Developing risk prediction algorithms including CSRE responses may enhance clinical evaluation.

## Key Points

**Question:**

Which elements of the nationally standardized Veterans Health Administration (VHA) Comprehensive Suicide Risk Evaluation (CSRE) are associated with subsequent suicide?

**Findings:**

In this cohort study, of the 269 374 CSREs completed by 153 736 VHA patients, suicidal ideation, firearm access, and preparatory behaviors were associated with acute (within 30 days) and chronic (within 365 days) suicide risk. In adjusted models, no protective factors (eg, hope, social support) in the CSRE were associated with acute or chronic suicide risk.

**Meaning:**

Findings suggest that VHA clinicians would benefit from a particular focus on a subset of CSRE responses, and that developing risk prediction algorithms may enhance clinical evaluations of suicide risk.

## Teaser

This cohort study evaluates the association of Comprehensive Suicide Risk Evaluation responses with suicide in a large, national cohort of patients evaluated within the Veterans Health Administration.

## Introduction

The age- and sex-adjusted suicide rate among US veterans increased by 76.3% from 2001 to 2021.^1^ Suicide prevention is the top clinical priority of the Department of Veterans Affairs (VA),^2^ and improving suicide risk detection among patients treated in the Veterans Health Administration (VHA) is a key component of a comprehensive approach to reducing suicide.^3^

The VHA implemented Risk ID, a standardized procedure for systemwide suicide screening and risk assessment, in 2018.^4^ The current version of Risk ID mandates that VHA patients receive annual suicide screening via the Columbia Suicide Severity Rating Scale (C-SSRS).^5^ Clinical reminders are integrated into the VHA electronic health record to notify clinicians when the screen is due. When a patient reports thinking about how they would attempt suicide in the past month or suicidal behavior in the prior 3 months (ie, positive screens), a licensed independent clinician is required to conduct the VHA’s Comprehensive Suicide Risk Evaluation (CSRE) to further assess suicidal ideation and behavior, suicide warning signs, risk factors, and protective factors.^6^ On the CSRE, warning signs signal an acute increase in risk of suicidal behavior in the immediate future. CSRE requirements are consistent across clinical settings, such that mental health and non–mental health clinicians may complete the CSRE. The CSRE can also be administered without first completing a C-SSRS. For example, mental health clinicians may routinely administer the CSRE as part of evaluations. Despite the national adoption of the CSRE, no study, to our knowledge, has examined the prospective associations between CSRE responses and suicide.

The CSRE was developed to include evidence-based factors associated with suicide^6^ as reflected by the VA–Department of Defense (DoD) Clinical Practice Guidelines^4^ and The Joint Commission’s National Patient Safety Goal, version 15.01.01.^7^ Although training materials are available, the CSRE is not a structured interview and does not provide question prompts. Based on their assessment, clinicians place patients in high-, intermediate-, or low-risk strata for acute and chronic suicide risk. Identifying CSRE responses associated with subsequent suicide could inform evidence-based suicide risk determinations. Establishing the incremental value of the CSRE after accounting for demographic and diagnostic risk factors within the clinical record may also inform current practices.

This study evaluated suicide in association with CSRE responses in a large national cohort of VHA patients, after adjusting for demographic factors and mental health diagnoses. Our primary analyses examined acute (within 30 days after CSRE administration) and chronic (within 365 days after CSRE administration) suicide risk.

## Methods

This population-based cohort study was conducted using methods approved by the Ann Arbor VA’s Institutional Review Board and under a waiver of informed consent. We followed Strengthening the Reporting of Observational Studies in Epidemiology (STROBE) guidelines for reporting cohort studies. Data collection and analysis were conducted from April 5 to August 20, 2024.

### Data Sources

The VHA Corporate Data Warehouse (CDW) was used to obtain demographic and clinical information. Vital status and cause of death were obtained via the VA-DoD Mortality Data Repository.^8^

### Cohort

Completed CSREs recorded in VHA records from November 1, 2019, through December 31, 2020, were included. November 1, 2019, was the initial inclusion date given the CSRE was implemented nationally on that date.^6^ Analyses treated CSREs as the unit of analysis, allowing VHA patients to have multiple evaluations. CSREs marked as invalid by the clinician and CSREs that were documented on or after the date of death were excluded.

### Measures

#### CSRE

The CSRE is a tool that specifies constructs for clinicians to use during suicide risk assessments. The CSRE includes 5 suicidal ideation, behavior, and lethal means items (ie, suicidal ideation, firearm access, nonfirearm lethal means access, prior suicide attempts, preparatory behavior), as well as 12 warning signs (eg, hopelessness), 12 risk factors (eg, recent losses), and 14 protective factors (eg, social support). CSRE items are scored on a binary presence or absence scale, with suicide-related items also including an option of unknown (combined with absence for these analyses). The CSRE does not include question prompts. Using clinical judgment, clinicians place patients in low-, intermediate-, and high-risk strata for acute and chronic suicide risk. CSRE responses are stored as health factors in the CDW.

#### C-SSRS Screen

The C-SSRS is a clinician-administered assessment that screens for suicidal ideation, planning, and behavior.^5^ Items are scored on a binary yes or no scale. In the VHA, thinking about how to make a suicide attempt in the past month and/or suicidal behavior within the past 3 months are positive screen indications. CSREs completed within 2 days of a C-SSRS (ie, those likely conducted following VA’s Risk ID protocols) were examined in a supplemental analysis. C-SSRS item responses were not included in these models.

#### Suicide Mortality

Suicides were identified by queries of the VA-DoD Mortality Data Repository, which includes results from annual VA-DoD searches of death certificates from the Centers for Disease Control and Prevention National Death Index.^8^ *International Statistical Classification of Diseases, Tenth Revision*,^9^ cause of death codes used to identify suicides included U03, X60 to X84, and Y87.0.

#### Covariates

Demographic factors identified via CDW based on prior VHA suicide studies^1^ included age, biological sex, race (American Indian or Alaska Native, Asian, Black, Native Hawaiian or Other Pacific Islander, White, multiracial, or unknown), ethnicity (Hispanic or non-Hispanic), and service connection. Psychiatric diagnoses were identified in the 365 days prior to CSRE administration. We also included the setting in which the CSRE was completed, categorized into emergency department, inpatient psychiatric, inpatient nonpsychiatric, outpatient mental health, outpatient non–mental health (eg, specialty medicine, non–mental health social work, urgent care), primary care, and primary care–mental health integration. The setting where the CSRE was completed may differ from the screening setting, as patients may be transferred from medicine clinics to mental health clinics to complete the CSRE.

### Statistical Analysis

Analyses were conducted in SAS Enterprise, version 9.4.^10^ Bivariate associations between study constructs and suicide in the 30 and 365 days following CSRE administration were examined. Next, multivariable Cox proportional hazards regression models were estimated, with risk time beginning the day following CSRE administration. Analyses adjusted for the clustering of CSREs within patients, allowing patients to contribute overlapping risk time for multiple CSREs, consistent with other VHA examinations.^11,12^ Multicollinearity diagnostics were examined, with correlation coefficients of 0.80 or greater, variance inflation factor values of 2.50 or greater, and tolerance values of 0.20 or less indicating problematic multicollinearity.^13^

Two Cox proportional hazards regression models examined the associations between CSRE responses and suicide. In the analysis of acute suicide risk, risk time continued for 30 days after CSRE administration or until death, whichever occurred first. Risk time for chronic suicide risk continued for 365 days or until death, whichever occurred first. Models were generated with and without clinician-determined risk strata. In the models without clinician-determined risk strata, the only substantive change was the hazard ratio (HR) for preexisting risk factors to 0.78 from 0.77, meaning it no longer met criteria for being statistically and clinically significant. We present the models with clinician-determined risk strata in the main text and models without clinician-determined risk strata in eTable 1 in Supplement 1.

Sensitivity analyses examined associations between CSRE responses and suicide for initial CSREs and for CSREs within 2 days of a positive C-SSRS screen (see eTables 2 and 3 in Supplement 1, respectively). We considered effect estimates to be statistically and clinically significant if HRs were 0.77 or less or 1.30 or greater with a 2-sided *P* ≤ .05.^14^

## Results

A total of 272 335 CSREs were completed from November 1, 2019, to December 31, 2020, with 269 374 (98.91%) marked valid and included in analyses (including 153 736 unique VHA patients). This includes 138 invalid CSREs that were completed on the same day as an invalid CSRE. Among the 269 374 CSREs, 86.26% were for male and 13.74% for female patients; 24.69% were for Black and 63.54% for White patients, with 7.22% of unknown race; and the mean (SD) age was 50.48 (15.26) years. Most patients completed a single CSRE during the period of interest (103 424 [67.27%]; 50 312 patients completed >1 CSRE; mean [SD] No. of CSREs, 1.75 [1.82]). Overall, 9107 CSREs (3.38%) were completed by individuals who died within 365 days of CSRE administration (mean [SD] risk time, 358.66 [39.01] days; median, 365.00 [1-365] days; total risk time, 96 613 410.00 days), with 791 CSREs (0.29%) completed by 385 individuals who died by suicide. The unadjusted suicide rate per 100 000 person-years following an initial CSRE was 228.92 (95% CI, 205.43-253.66). Bivariate associations between suicide and covariates can be found in Table 1. In bivariate analyses, 24 of the 43 CSRE constructs (55.81%) were associated with suicide within 30 or 365 days, with 10 of the 43 CSRE responses (23.26%) associated with suicide within 30 days after the CSRE, and 23 (53.49%) were associated with suicide within 365 days (Table 2). Patients at high acute risk (as identified by their clinician) were at greater suicide risk within 30 days (HR, 3.21; 95% CI, 1.99-5.17; *P* < .001) and 365 days (HR, 2.72; 95% CI, 2.14-3.46; *P* < .001) compared with patients at low acute risk, and patients at high chronic risk were at greater suicide risk within 30 days (HR, 3.82; 95% CI, 2.16-6.73; *P* < .001) and 365 days (HR, 3.89; 95% CI, 2.92-5.19; *P* < .001), compared with patients at low chronic risk. However, 172 CSREs for 133 unique suicide decedents were assessed as being at low acute and low chronic risk. We also examined clinician-determined acute and chronic risk strata combinations, finding that patients with low acute and low chronic risks had the lowest subsequent suicide risk. Suicide risk increased with each tier of clinician-determined risk strata (eTable 4 in Supplement 1).

**Table 1.** Demographic and Psychiatric Diagnosis Characteristics^a^

Variable	Suicide within 30 d				Suicide within 365 d			
	No. (%) of CSREs		HR (95% CI)	*P* value	No. (%) of CSREs		HR (95% CI)	*P* value
	Present (n = 144)	Absent (n = 269 230)			Present (n = 791)	Absent (n = 268 583)		
Age, y								
18-34	22 (15.28)	51 445 (19.11)	1 [Reference]	NA	193 (24.40)	51 274 (19.09)	1 [Reference]	NA
35-54	55 (38.19)	99 474 (36.95)	1.29 (0.59-2.82)	.52	284 (35.90)	99 245 (36.95)	0.76 (0.55-1.06)	.11
≥55	67 (46.53)	118 309 (43.94)	1.33 (0.63-2.78)	.46	314 (39.70)	118 062 (43.96)	0.72 (0.51-1.03)	.07
Sex								
Female	19 (13.19)	36 982 (13.74)	0.96 (0.41-2.21)	.91	73 (9.23)	36 928 (13.75)	0.63 (0.41-0.99)	.04
Male	125 (86.81)	232 248 (86.26)	1 [Reference]	NA	718 (90.77)	231 655 (86.25)	1 [Reference]	NA
Race								
American Indian or Alaska Native	<10	<10	NA	NA	<10	<10	NA	NA
Asian	<10	<10	NA	NA	<10	<10	NA	NA
Black	10 (6.94)	66 485 (24.69)	0.23 (0.11-0.51)	<.001	50 (6.32)	66 445 (24.74)	0.20 (0.13-0.30)	<.001
Native Hawaiian or Other Pacific Islander	<10	<10	NA	NA	<10	<10	NA	NA
White	110 (76.39)	171 077 (63.54)	1 [Reference]	NA	651 (82.30)	170 536 (63.49)	1 [Reference]	NA
Multiracial	<10	<10	NA	NA	<10	<10	NA	NA
Unknown	17 (11.81)	19 444 (7.22)	1.36 (0.67-2.77)	.40	51 (6.45)	19 410 (7.23)	0.69 (0.44-1.07)	.10
Hispanic ethnicity	<10	<10	NA	NA	52 (6.57)	22 288 (8.30)	0.77 (0.47-1.28)	.31
Service connected	66 (45.83)	198 271 (73.64)	0.30 (0.19-0.49)	<.001	454 (57.40)	197 883 (73.68)	0.48 (0.36-0.63)	<.001
CSRE setting								
ED	12 (8.33)	11 891 (4.42)	2.07 (1.05-4.07)	.04	50 (6.32)	11 853 (4.41)	1.54 (1.13-2.10)	.007
IP MH	<10	<10	NA	NA	<10	<10	NA	NA
IP non-MH	<10	<10	NA	NA	<10	<10	NA	NA
OP MH	98 (68.06)	200 485 (74.47)	1 [Reference]	NA	553 (69.91)	200 030 (74.48)	1 [Reference]	NA
OP non-MH	30 (20.83)	44 997 (16.71)	1.37 (0.88-2.12)	.17	158 (19.97)	44 869 (16.71)	1.28 (1.02-1.62)	.03
Primary care	<10	<10	NA	NA	<10	<10	NA	NA
PCMHI	<10	<10	NA	NA	14 (1.77)	8050 (3.00)	0.63 (0.35-1.11)	.11
Mental health diagnosis								
Depressive disorder	100 (69.44)	184 659 (68.59)	1.04 (0.68-1.60)	.86	597 (75.47)	184 162 (68.57)	1.41 (1.11-1.79)	.005
Bipolar disorder	35 (24.31)	48 936 (18.18)	1.45 (0.81-2.59)	.21	222 (28.07)	48 749 (18.15)	1.76 (1.28-2.41)	<.001
Schizophrenia spectrum disorder	24 (16.67)	28 729 (10.67)	1.67 (0.78-3.62)	.19	127 (16.06)	28 626 (10.66)	1.61 (1.06-2.44)	.03
PTSD	62 (43.06)	142 700 (53.00)	0.67 (0.41-1.10)	.11	373 (47.16)	142 389 (53.01)	0.79 (0.61-1.02)	.07
Anxiety disorder	75 (52.08)	118 948 (44.18)	1.37 (0.86-2.19)	.18	469 (59.29)	118 554 (44.14)	1.84 (1.44-2.36)	<.001
AUD and/or SUD	82 (56.94)	149 724 (55.61)	1.06 (0.66-1.68)	.82	517 (65.36)	149 289 (55.58)	1.51 (1.16-1.97)	.003
Insomnia disorder	43 (29.86)	69 925 (25.97)	1.21 (0.70-2.10)	.49	246 (31.10)	69 722 (25.96)	1.29 (0.97-1.72)	.08

Abbreviations: AUD, alcohol use disorder; CSRE, Comprehensive Suicide Risk Evaluation; ED, emergency department; HR, hazard ratio; IP, inpatient; MH, mental health; NA, not applicable; OP, outpatient; PCMHI, primary care–mental health integration; PTSD, posttraumatic stress disorder; SUD, substance use disorder.

^a^ Includes data from 269 374 valid CSREs from 153 736 patients. Cell sizes of less than 10 patients are suppressed per privacy guidelines. Numbers may not sum to totals in column headings owing to missing data.

**Table 2.** Characteristics of Unique CSRE Administrations^a^

Variable	Suicide within 30 d					Suicide within 365 d				
	No. (%) of CSREs		HR (95% CI)	*P* value	No. (%) of CSREs			HR (95% CI)	*P* value
	Present (n = 144)	Absent (n = 269 230)			Present (n = 791)		Absent (n = 268 583)		
CSRE									
Suicidal ideation	128 (88.89)	180 285 (66.96)	3.94 (2.19-7.09)	<.001	672 (84.96)		179 741 (66.92)	2.78 (2.13-3.63)	<.001
Firearm access	35 (24.31)	44 283 (16.45)	1.63 (1.04-2.56)	.03	149 (18.84)		44 169 (16.44)	1.17 (0.90-1.53)	.24
Other lethal means access	38 (26.39)	44 105 (16.38)	1.83 (1.19-2.82)	.006	179 (22.63)		43 964 (16.37)	1.49 (1.19-1.87)	<.001
Prior suicide attempt	16 (11.11)	33 488 (12.44)	0.88 (0.51-1.51)	.64	133 (16.81)		33 371 (12.42)	1.42 (1.16-1.74)	<.001
Preparatory behavior	16 (11.11)	11 703 (4.35)	2.75 (1.64-4.60)	<.001	59 (7.46)		11 660 (4.34)	1.77 (1.34-2.32)	<.001
Warning signs									
Suicidal communication	40 (27.78)	35 915 (13.34)	2.50 (1.75-3.57)	<.001	182 (23.01)		35 773 (13.32)	1.95 (1.61-2.36)	<.001
Direct preparations	23 (15.97)	10 905 (4.05)	4.50 (2.94-6.89)	<.001	85 (10.75)		10 843 (4.04)	2.85 (2.12-3.82)	<.001
Seeking lethal means access	21 (14.58)	10 442 (3.88)	4.23 (2.65-6.75)	<.001	74 (9.36)		10 389 (3.87)	2.56 (1.96-3.34)	<.001
Anger	19 (13.19)	44 491 (16.53)	0.77 (0.46-1.28)	.31	112 (14.16)		44 398 (16.53)	0.83 (0.67-1.04)	.10
Anxiety	61 (42.36)	77 665 (28.85)	1.81 (1.24-2.65)	.002	294 (37.17)		77 432 (28.83)	1.45 (1.22-1.73)	<.001
Guilt	31 (21.53)	44 050 (16.36)	1.40 (0.90-2.18)	.13	174 (22.00)		43 907 (16.35)	1.43 (1.18-1.74)	<.001
Hopelessness	48 (33.33)	64 277 (23.87)	1.60 (1.09-2.34)	.02	269 (34.01)		64 056 (23.85)	1.64 (1.38-1.97)	<.001
Increased isolation	29 (20.14)	46 711 (17.35)	1.20 (0.75-1.93)	.45	176 (22.25)		46 564 (17.34)	1.36 (1.11-1.67)	.004
Reckless behaviors	14 (9.72)	16 711 (6.21)	1.63 (0.99-2.67)	.06	87 (11.00)		16 638 (6.19)	1.87 (1.41-2.46)	<.001
Sleep disturbance	36 (25.00)	65 061 (24.17)	1.05 (0.70-1.56)	.82	216 (27.31)		64 881 (24.16)	1.18 (0.99-1.40)	.07
Substance abuse	23 (15.97)	39 018 (14.49)	1.12 (0.73-1.72)	.60	157 (19.85)		38 884 (14.48)	1.46 (1.18-1.82)	<.001
Other	17 (11.81)	20 772 (7.72)	1.60 (0.95-2.71)	.08	94 (11.88)		20 695 (7.71)	1.62 (1.29-2.03)	<.001
Risk factors									
History of suicidal behavior	69 (47.92)	105 934 (39.35)	1.42 (0.92-2.18)	.11	437 (55.25)		105 566 (39.30)	1.90 (1.52-2.38)	<.001
Recent stressor	95 (65.97)	151 533 (56.28)	1.51 (0.98-2.31)	.06	499 (63.08)		151 129 (56.27)	1.32 (1.07-1.63)	.009
Access to lethal means	45 (31.25)	64 474 (23.95)	1.44 (0.99-2.10)	.06	240 (30.34)		64 279 (23.93)	1.38 (1.13-1.69)	.002
History of psychiatric hospitalization	90 (62.50)	124 494 (46.24)	1.94 (1.30-2.88)	.001	530 (67.00)		124 054 (46.19)	2.36 (1.93-2.89)	<.001
Psychiatric conditions	107 (74.31)	197 821 (73.48)	1.04 (0.69-1.59)	.85	624 (78.89)		197 304 (73.46)	1.34 (1.10-1.64)	.004
History of nonsuicidal self-injury	11 (7.64)	17 705 (6.58)	1.17 (0.60-2.30)	.64	77 (9.73)		17 639 (6.57)	1.52 (1.09-2.13)	.01
Recent losses	32 (22.22)	55 359 (20.56)	1.10 (0.70-1.74)	.67	174 (22.00)		55 217 (20.56)	1.09 (0.87-1.36)	.46
Preexisting risk factors (eg, trauma)	34 (23.61)	85 249 (31.66)	0.67 (0.44-1.01)	.06	221 (27.94)		85 062 (31.67)	0.83 (0.68-1.02)	.08
Chronic medical condition	57 (39.58)	98 559 (36.61)	1.14 (0.73-1.76)	.57	310 (39.19)		98 306 (36.60)	1.13 (0.93-1.37)	.23
Marginalized group status	<10	<10	NA	NA	32 (4.05)		11 239 (4.18)	0.96 (0.52-1.77)	.91
Recent transition from military	<10	<10	NA	NA	12 (1.52)		8603 (3.20)	0.46 (0.26-0.82)	.008
Other	<10	<10	NA	NA	57 (7.21)		15 060 (5.61)	1.31 (0.90-1.91)	.16
Protective factor									
Access to health care	92 (63.89)	179 710 (66.75)	0.88 (0.60-1.30)	.53	535 (67.64)		179 267 (66.75)	1.04 (0.87-1.25)	.65
Motivation for medical treatment	64 (44.44)	134 690 (50.03)	0.80 (0.56-1.14)	.22	406 (51.33)		134 348 (50.02)	1.06 (0.89-1.26)	.54
Access to MH care	93 (64.58)	177 848 (66.06)	0.94 (0.62-1.41)	.75	541 (68.39)		177 400 (66.05)	1.11 (0.92-1.33)	.28
Motivation for MH treatment	88 (61.11)	181 066 (67.25)	0.76 (0.54-1.07)	.12	551 (69.66)		180 603 (67.24)	1.11 (0.91-1.35)	.30
Positive interpersonal relationships	93 (64.58)	165 322 (61.41)	1.15 (0.78-1.68)	.48	473 (59.80)		164 942 (61.41)	0.93 (0.77-1.13)	.47
Significant other	41 (28.47)	90 674 (33.68)	0.78 (0.48-1.27)	.32	254 (32.11)		90 461 (33.68)	0.93 (0.72-1.19)	.55
Caregiving responsibilities	35 (24.31)	70 774 (26.29)	0.90 (0.54-1.51)	.69	175 (22.12)		70 634 (26.30)	0.79 (0.61-1.03)	.08
Hope for the future	72 (50.00)	141 487 (52.55)	0.90 (0.62-1.31)	.59	398 (50.32)		141 161 (52.56)	0.91 (0.77-1.08)	.28
Personal traits or beliefs against suicide	55 (38.19)	109 678 (40.74)	0.90 (0.62-1.30)	.57	277 (35.02)		109 456 (40.75)	0.78 (0.65-0.94)^b^	.01
Religious beliefs against suicide	31 (21.53)	73 896 (27.45)	0.73 (0.43-1.23)	.23	173 (21.87)		73 754 (27.46)	0.74 (0.57-0.96)	.02
Cultural connections	<10	<10	NA	NA	43 (5.44)		22 606 (8.42)	0.63 (0.44-0.90)	.01
Social support	39 (27.08)	80 821 (30.02)	0.87 (0.60-1.26)	.45	214 (27.05)		80 646 (30.03)	0.86 (0.71-1.05)	.14
Desire to live	51 (35.42)	107 080 (39.77)	0.83 (0.53-1.29)	.41	270 (34.13)		106 861 (39.79)	0.78 (0.64-0.95)^b^	.01
Other	<10	<10	NA	NA	49 (6.19)		11 801 (4.39)	1.44 (0.98-2.11)	.06
Acute suicide risk				<.001					<.001
Low	73 (50.69)	186 670 (69.38)	1 [Reference]	NA	399 (50.44)		186 344 (69.42)	1 [Reference]	NA
Intermediate	47 (32.64)	63 235 (23.50)	1.90 (1.28-2.83)	.002	281 (35.52)		63 001 (23.47)	2.08 (1.73-2.49)	<.001
High	24 (16.67)	19 159 (7.12)	3.21 (1.99-5.17)	<.001	111 (14.03)		19 072 (7.11)	2.72 (2.14-3.46)	<.001
Chronic suicide risk				<.001					<.001
Low	39 (27.08)	126 711 (47.09)	1 [Reference]	NA	203 (25.66)		126 547 (47.15)	1 [Reference]	NA
Intermediate	75 (52.08)	116 810 (43.41)	2.09 (1.33-3.27)	.001	429 (54.24)		116 456 (43.39)	2.29 (1.82-2.89)	<.001
High	30 (20.83)	25 539 (9.49)	3.82 (2.16-6.73)	<.001	159 (20.10)		25 410 (9.47)	3.89 (2.92-5.19)	<.001

Abbreviations: CSRE, Comprehensive Suicide Risk Evaluation; HR, hazard ratio; MH, mental health; MHC, mental health clinic; NA, not applicable.

^a^ Includes data from 269 374 valid CSREs from 153 736 patients. Cell sizes less than 10 patients are suppressed per privacy guidelines. Numbers may not sum to totals in column headings owing to missing data. Numbers may not sum to totals in column headings owing to missing data.

^b^ Effect did not meet criteria for being statistically and clinically significant (ie, HR ≤0.77 or ≥1.30 [*P* ≤ .05]).

### Suicide in the 30 Days After CSRE Administration

Of the 791 suicides in the 365 days following a CSRE, 144 occurred within 30 days (18.20%). No variables evidenced problematic multicollinearity based on bivariate correlations, variance inflation factors, or tolerance values, and the proportional hazards assumption held for patients with and without suicidal ideation on the CSRE. Increased suicide risk within 30 days of CSRE administration was found for CSREs with suicidal ideation (HR, 3.14; 95% CI, 1.51-6.54) (Table 3), firearm access (HR, 2.62; 95% CI, 1.49-4.61), direct preparations for a suicide attempt (HR, 2.15; 95% CI, 1.27-3.62), seeking access to lethal means (HR, 2.04; 95% CI, 1.11-3.75), anxiety (HR, 1.80; 95% CI, 1.16-2.81), and history of psychiatric hospitalization (HR, 1.63; 95% CI, 1.02-2.61). Anger (HR, 0.50; 95% CI, 0.30-0.85) was associated with decreased suicide risk within 30 days of CSRE administration.

**Table 3.** Cox Proportional Hazards Regression Models Examining Associations With Suicide Within 30 and 365 Days of CSRE Administration

Predictive factor	Suicide within 30 d (n = 269 196)		Suicide within 365 d (n = 269 196)	
	HR (95% CI)	*P* value	HR (95% CI)	*P* value
Age, y				
18-34	1 [Reference]	NA	1 [Reference]	NA
35-54	1.33 (0.62-2.86)	.47	0.73 (0.52-1.02)	.07
≥55	1.18 (0.56-2.46)	.67	0.60 (0.40-0.91)	.02
Sex				
Female	1.17 (0.51-2.70)	.71	0.71 (0.45-1.11)	.13
Male	1 [Reference]	NA	1 [Reference]	NA
Race				
American Indian or Alaska Native	0.62 (0.08-4.61)	.64	1.11 (0.40-3.11)	.84
Asian	4.22 (0.65-27.24)	.13	1.45 (0.35-5.95)	.61
Black	0.28 (0.12-0.62)	.002	0.23 (0.15-0.36)	<.001
Native Hawaiian or Other Pacific Islander	NA^b^	NA	0.50 (0.11-2.37)	.38
White	1 [Reference]	NA	1 [Reference]	NA
Multiracial	NA^b^	NA	0.75 (0.15-3.72)	.72
Unknown	1.54 (0.73-3.24)	.26	0.75 (0.48-1.17)	.21
Hispanic ethnicity	0.24 (0.08-0.79)	.02	0.74 (0.45-1.22)	.23
Service connection	0.32 (0.19-0.54)	<.001	0.48 (0.35-0.67)	<.001
Setting				
ED	1.40 (0.68-2.88)	.36	1.09 (0.78-1.52)	.62
IP MH	1.88 (0.44-8.13)	.40	1.53 (0.73-3.19)	.26
IP non-MH	NA^b^	NA	1.97 (0.49-7.99)	.34
OP non-MH	1.10 (0.72-1.70)	.66	1.08 (0.85-1.37)	.54
Primary care	0.84 (0.11-6.32)	.87	1.00 (0.41-2.44)	.99
PCMHI	0.23 (0.03-1.72)	.15	0.65 (0.36-1.16)	.14
MHC	1 [Reference]	NA	1 [Reference]	NA
Depressive disorder	0.81 (0.50-1.31)	.39	1.03 (0.80-1.33)	.83
Bipolar disorder	1.07 (0.61-1.89)	.81	1.22 (0.89-1.69)	.22
Schizophrenia spectrum disorder	1.51 (0.67-3.42)	.32	1.37 (0.86-2.19)	.18
PTSD	0.86 (0.56-1.31)	.47	0.75 (0.58-0.98)	.03
Anxiety disorder	1.21 (0.74-1.97)	.46	1.45 (1.12-1.89)	.005
AUD and/or SUD	0.93 (0.55-1.57)	.78	1.14 (0.84-1.55)	.40
Insomnia disorder	1.09 (0.61-1.95)	.77	1.01 (0.76-1.35)	.95
CSRE				
Suicidal ideation	3.14 (1.51-6.54)	.002	1.63 (1.20-2.21)	.002
Firearm access	2.62 (1.49-4.61)	<.001	1.55 (1.13-2.13)	.006
Other lethal means access	1.37 (0.88-2.13)	.16	0.98 (0.78-1.22)	.84
Prior suicide attempt	0.59 (0.33-1.04)	.07	1.00 (0.80-1.26)	.98
Preparatory behavior	1.56 (0.89-2.74)	.12	1.11 (0.83-1.49)	.47
Warning signs				
Suicidal communication	1.28 (0.82-2.00)	.28	1.13 (0.90-1.42)	.30
Direct preparations	2.15 (1.27-3.62)	.004	1.56 (1.09-2.23)	.02
Seeking lethal means access	2.04 (1.11-3.75)	.02	1.23 (0.88-1.72)	.23
Anger	0.50 (0.30-0.85)	.01	0.56 (0.44-0.71)	<.001
Anxiety	1.80 (1.16-2.81)	.009	1.14 (0.92-1.41)	.23
Guilt	1.11 (0.68-1.82)	.68	1.12 (0.90-1.38)	.32
Hopelessness	0.86 (0.52-1.41)	.54	1.04 (0.84-1.29)	.72
Increased isolation	0.83 (0.48-1.42)	.49	0.98 (0.78-1.23)	.85
Reckless behaviors	1.32 (0.77-2.26)	.31	1.40 (1.00-1.95)	.05
Sleep disturbance	0.70 (0.42-1.17)	.17	0.87 (0.70-1.07)	.18
Substance abuse	0.70 (0.42-1.16)	.17	0.94 (0.73-1.21)	.63
Other	1.12 (0.66-1.91)	.66	1.23 (0.98-1.54)	.07
Risk factors				
History of suicidal behavior	0.90 (0.56-1.47)	.68	1.05 (0.80-1.38)	.71
Recent stressor	1.15 (0.75-1.76)	.52	0.96 (0.76-1.20)	.71
Access to lethal means	0.64 (0.39-1.05)	.08	0.93 (0.72-1.20)	.57
History of psychiatric hospitalization	1.63 (1.02-2.61)	.04	1.68 (1.32-2.13)	<.001
Psychiatric conditions	0.82 (0.52-1.28)	.38	0.99 (0.80-1.22)	.93
History of nonsuicidal self-injury	0.94 (0.51-1.73)	.84	1.03 (0.74-1.44)	.86
Recent losses	0.97 (0.62-1.53)	.91	0.97 (0.77-1.22)	.79
Preexisting risk factors (eg, trauma)	0.66 (0.41-1.04)	.07	0.77 (0.62-0.96)	.02
Chronic medical condition	1.19 (0.75-1.90)	.47	1.17 (0.94-1.46)	.16
Marginalized group status	0.34 (0.08-1.44)	.14	1.02 (0.54-1.93)	.96
Recent transition from military	0.47 (0.11-2.04)	.31	0.39 (0.22-0.70)	.002
Other	0.89 (0.40-1.97)	.77	1.23 (0.86-1.75)	.26
Protective factors				
Access to health care	0.96 (0.59-1.57)	.88	1.06 (0.87-1.29)	.56
Motivation for medical treatment	0.85 (0.55-1.31)	.47	1.09 (0.91-1.30)	.35
Access to MH care	0.96 (0.62-1.49)	.86	0.97 (0.80-1.17)	.75
Motivation for MH treatment	0.73 (0.51-1.05)	.09	1.01 (0.83-1.23)	.92
Positive interpersonal relationships	1.52 (0.98-2.35)	.06	1.14 (0.94-1.39)	.18
Significant other	0.88 (0.50-1.53)	.64	1.15 (0.87-1.51)	.32
Caregiving responsibilities	1.08 (0.59-1.98)	.80	0.87 (0.65-1.17)	.35
Hope for the future	1.18 (0.78-1.79)	.44	1.10 (0.91-1.32)	.34
Personal traits or beliefs against suicide	1.03 (0.66-1.59)	.90	0.81 (0.67-0.99)^a^	.04
Religious beliefs against suicide	0.81 (0.48-1.37)	.44	0.92 (0.71-1.17)	.48
Cultural connections	1.02 (0.58-1.80)	.94	0.84 (0.61-1.17)	.30
Social support	0.94 (0.64-1.39)	.76	0.95 (0.77-1.17)	.62
Desire to live	1.20 (0.74-1.93)	.47	1.02 (0.83-1.26)	.87
Other	1.13 (0.57-2.25)	.73	1.20 (0.83-1.74)	.33
Acute suicide risk				
High	1.18 (0.60-2.35)	.63	1.27 (0.92-1.75)	.15
Intermediate	1.19 (0.77-1.84)	.45	1.39 (1.13-1.71)	.002
Low	1 [Reference]	NA	1 [Reference]	NA
Chronic suicide risk				
High	1.88 (0.92-3.85)	.08	1.74 (1.22-2.47)	.002
Intermediate	1.32 (0.76-2.30)	.32	1.34 (1.01-1.77)	.04
Low	1 [Reference]	NA	1 [Reference]	NA

Abbreviations: AUD, alcohol use disorder; CSRE, comprehensive suicide risk evaluation; ED, emergency department; HR, hazard ratio; IP, inpatient; MH, mental health; MHC, mental health clinic; NA, not applicable; OP, outpatient; PCMHI, primary care–mental health integration; PTSD, posttraumatic stress disorder; SUD, substance use disorder.

^a^ Effect did not meet criteria for being statistically and clinically significant (ie, HR ≤0.77 or ≥1.30 [*P* ≤ .05]).

^b^ Effect estimates based on cell sizes of less than 10 patients are suppressed per privacy guidelines.

### Suicide in the 365 Days After CSRE Administration

Suicidal ideation (HR, 1.63; 95% CI, 1.20-2.21) (Table 3), firearm access (HR, 1.55; 95% CI, 1.13-2.13), direct suicide preparations (HR, 1.56; 95% CI, 1.09-2.23), reckless behaviors (HR, 1.40; 95% CI, 1.00-1.95]), and history of psychiatric hospitalization (HR, 1.68; 95% CI, 1.32-2.13) were associated with a significantly increased suicide risk in the 365 days following CSRE administration after controlling for demographic factors, setting, and psychiatric diagnoses, whereas anger (HR, 0.56; 95% CI, 0.44-0.71), preexisting risk factors (eg, trauma; HR, 0.77; 95% CI, 0.62-0.96), and recent transition from the military (HR, 0.39; 95% CI, 0.22-0.70) were associated with decreased suicide risk. Clinician-reported risk assessment ratings associated with increased suicide risk were intermediate acute risk (HR, 1.39; 95% CI, 1.13-1.71), intermediate chronic risk (HR, 1.34; 95% CI, 1.01-1.77), and high chronic risk (HR, 1.74; 95% CI, 1.22-2.47), compared with low risk.

## Discussion

Models of the 43 constructs included in the nationally standardized VHA CSRE identified only 5 that found clinically and statistically significant associations with suicide within both 30 days and 365 days. These constructs include suicidal ideation, firearm access, preparatory behaviors, and psychiatric hospitalization history as risk factors, whereas anger was unexpectedly associated with reduced risk. The CSRE includes 14 constructs it identifies as protective factors, yet none of these factors were associated with suicide in adjusted models. Taken together, findings indicate that a narrow set of risk factors—namely access and preparations to use lethal means—are most critical when assessing acute and chronic suicide risk; in the presence of these risk factors, previously identified protective factors may be insufficient to prevent suicide.

Anxiety was additionally associated with suicide within 30 days of a CSRE, which is consistent with studies of acute warning signs of suicide attempts^15,16^ and negative reinforcement models of suicide.^17,18^ Reckless behaviors were also associated with suicide within 365 days, as consistent with extant research.^19^ Prior studies also reported alcohol use as a warning sign for suicide attempts,^15,20,21^ whereas substance use (including alcohol) on the CSRE was not associated with suicide. This contrast in findings could be due to the CSRE not isolating alcohol-specific risk or could reflect differences in warning signs for suicide attempts vs suicide. Prior studies also identified interpersonal negative life events, perceived burdensomeness, and emptiness as warning signs for suicide attempts^15^; however, the CSRE does not explicitly include these domains.

Firearm access had a consistent positive association with suicide across all risk periods. Among veterans, firearm suicide rates exceed all other method-specific rates.^1^ The VHA has invested in interventions to limit firearm access during suicide crises, such as distributing gun locks.^4^ However, veterans who died by suicide via firearms are less likely to have received lethal means safety counseling and had lower levels of suicide risk factors (eg, prior suicide attempts),^22^ potentially due to the high lethality of firearms,^23,24,25^ indicating that they may be less likely to receive safety counseling. The consistency of these findings underscores the importance of reducing firearm access during suicide crises and expanding access to lethal means safety interventions.^26,27^

The CSRE includes 14 identified protective factors, yet none were associated with suicide in adjusted models. Protective factors for suicide attempts have been identified among veterans,^28,29,30^ such as social support. However, protective factors for suicide likely differ from suicide attempts, with literature suggesting social disconnection may protect against suicide attempts but not suicide.^31^ Research is needed to determine whether protective factors in the CSRE are associated with suicide attempts to support their continued inclusion in the CSRE and inform policies, such as The Joint Commission’s National Patient Safety Goals, that require an assessment of protective factors.^7^

Although 24 of the 43 CSRE constructs (55.81%) were associated with suicide in bivariate analyses, only 10 (23.26%) were associated with acute or chronic suicide risk in adjusted models. Clinicians report concern regarding the time required to conduct the CSRE.^32^ Reducing the CSRE to include only constructs associated with suicide or other relevant outcomes (eg, suicide attempts, hospitalization) could reduce patient and clinician burden, which may be particularly salient given competing demands during suicide crises.

Although assessments of intermediate acute and intermediate to high chronic risk were associated with suicide within 365 days, no clinician risk determinations were associated with suicide within 30 days in adjusted models. Additionally, 172 CSREs for 133 unique suicide decedents were assessed as being at low acute and low chronic risk. These findings suggest clinician judgments informed by the CSRE are imperfect predictive factors associated with suicide. This could be due to clinicians undervaluing risk factors like firearm access or overvaluing constructs with null associations with suicide during risk determinations. Research should explore which constructs are associated with suicide vs clinician risk determinations to identify potential opportunities for clinician education. Algorithmic feedback based on CSRE responses may hold promise for improving decision making for frontline providers.

There were unexpected findings. We found decreased chronic suicide risk for patients with anger, preexisting risk factors, and a recent transition from the military. Studies have found these constructs to be associated with increased suicide risk and related outcomes.^33,34,35,36^ In the VHA, anger is commonly associated with posttraumatic stress disorder (PTSD),^37,38,39^ and PTSD diagnoses were associated with decreased chronic suicide risk in this study. The VHA has implemented a national rollout of evidence-based PTSD treatments,^40^ and initiating evidence-based PTSD treatment is associated with reduced suicide risk in VHA.^41^ Treatment receipt following the CSRE could influence the association between anger and suicide, and future studies should consider PTSD diagnosis and treatment as possible moderators. Further, preexisting risk factors were associated with reduced suicide risk. The broad nature of this construct, combined with limited CSRE guidance (currently, the only examples are trauma and history of suicide attempt) suggests this association should be interpreted with caution. Finally, this study found reduced suicide risk associated with recent transition from the military. This is inconsistent with literature that identified the period following military separation as a “deadly gap” where former service members are at increased suicide risk.^33^ However, studies have typically included all US military personal,^42^ rather than those engaged in VHA care. Given that health care disruptions may contribute to this high-risk period,^43^ reduced suicide risk observed herein may be due to early engagement in VHA care.

### Limitations

This study has limitations. Conclusions about association between specific constructs and suicide are limited by the lack of standardized prompts and nonspecific nature of constructs included in the CSRE. For example, recent stressors could include myriad types of stressors. Therefore, CSRE responses should be interpreted as clinician-identified markers, rather than standardized items. Further, the CSRE may be improved by including constructs with strong evidence for predictive factors associated with suicidal ideation and behavior (eg, perceived burdensomeness^15,44,45,46^), or refining broad constructs such as substance use, where evidence suggests substance class is differentially associated with suicidal behavior.^47^ Notably, the CSRE is an evaluation tool designed to guide clinical assessment, not a validated risk prediction instrument, and these findings highlight the challenges of translating clinical evaluations into accurate suicide risk prediction. When interpreting findings, it is important to remember that the CSRE is only administered to a subset of VHA patients, likely those identified as at high risk. Highlighting the nonrepresentative nature of this sample, the unadjusted suicide rate per 100 000 person-years for initial CSREs was 228.92, far surpassing the Veteran suicide rate of 33.9.^1^ Therefore, results from this cohort may not extend to the overall VHA patient population or to populations outside VHA, and factors not associated with suicide among those who completed CSREs may still be associated with suicide in other populations. Additionally, this study used death certificate data, which may not capture all suicides.^48^ Further, it is possible that certain risk factors were underreported due to stigma or desire to avoid possible consequences (eg, hospitalization).^49,50^ Last, analyses were potentially confounded by subsequent treatment receipt, with patients identified as at high risk potentially being more likely to receive higher-care intensity, including hospitalization where access to lethal means is highly restricted. It may be that CSRE responses that were unexpectedly associated with decreased suicide risk also contributed to patients being identified as at high risk and receiving more intensive treatment.

## Conclusions

This cohort study provides, to our knowledge, the first national data regarding suicide among VHA patients following CSRE administration. Models of acute (within 30 days) and chronic (within 365 days) suicide risk highlight constructs that may be most relevant for clinical assessment of both short- and long-term risk of suicide. Further, the protective factors included in the CSRE were not associated with suicide, suggesting CSRE refinements may be warranted to minimize patient and clinician burden during suicide crises. Findings suggest VHA clinicians may benefit from a focus on a subset of CSRE responses when evaluating suicide risk. Refining the CSRE to focus on factors with the strongest associations with suicide, standardizing construct definitions, and developing risk prediction algorithms could potentially enhance clinical evaluation.

## Notes

Supplement 1. **eTable 1.** Cox Proportional Hazards Regression Models Examining Associations With Suicide Within 30 and 365 Days of CSRE Administration Without Clinician-Determined Risk Strata (Ns=269 372) **eTable 2.** Cox Proportional Hazards Regression Models Examining Predictors of Suicide in the Year After CSRE Administrations for Initial CSREs (Ns=153 648) **eTable 3.** Cox Proportional Hazards Regression Models Examining Predictors of Suicide in the Year After CSRE Administration for CSREs Within 2 Days of a Positive Columbia Suicide Severity Rating Scale Screen (Ns=81 102) **eTable 4.** Clinician-Determined Risk Strata Combinations and Suicide Following CSRE Receipt (Ns=269 198)

Supplement 2.
Data Sharing Statement
