# Errors in Discussion

**DOI:** 10.1001/jamanetworkopen.2025.8587

**Published:** 2025-03-28

In the Original Investigation titled “Suicide Risk Evaluations and Suicide in the Veterans Health Administration,”^1^ published February 25, 2025, there were errors in the Discussion. In the first sentence, the phrase “identified only 4” should have been “identified only 5.” The second sentence should have included psychiatric hospitalization history as a risk factor to read: “These constructs include suicidal ideation, firearm access, preparatory behaviors, and psychiatric hospitalization history as risk factors, whereas anger was unexpectedly associated with reduced risk.” This article has been corrected.^1^
