# Supplementary material for: Suicide Risk Evaluations and Suicide in the Veterans Health Administration
Source: JAMA Netw Open. 2025 Feb 25;8(2):e2461559. doi: 10.1001/jamanetworkopen.2024.61559 (PMC11862973; doi:10.1001/jamanetworkopen.2024.61559)
Supplement: Supplement 1. — eTable 1. Cox Proportional Hazards Regression Models Examining Associations With Suicide Within 30 and 365 Days of CSRE Administration Without Clinician-Determined Risk Strata (Ns=269 372) eTable 2. Cox Proportional Hazards Regression Models Examining Predictors of Suicide in the Year After CSRE Administrations for Initial CSREs (Ns=153 648) eTable 3. Cox Proportional Hazards Regression Models Examining Predictors of Suicide in the Year After CSRE Administration for CSREs Within 2 Days of a Positive Columbia Suicide Severity Rating Scale Screen (Ns=81 102) eTable 4. Clinician-Determined Risk Strata Combinations and Suicide Following CSRE Receipt (Ns=269 198) [file jamanetwopen-e2461559-s001.pdf]

Supplementary Online Content

Saulnier KG, Bagge CL, Ganoczy D, et al. Suicide risk evaluations and suicide in the Veterans Health Administration. *JAMA Netw Open*. 2025;8(2):e2461559. doi:10.1001/jamanetworkopen.2024.61559

eTable 1. Cox Proportional Hazards Regression Models Examining Associations With Suicide Within 30 and 365 Days of CSRE Administration Without Clinician-Determined Risk Strata (Ns = 269 372)
eTable 2. Cox Proportional Hazards Regression Models Examining Predictors of Suicide in the Year After CSRE Administrations for Initial CSREs (Ns = 153 648)
eTable 3. Cox Proportional Hazards Regression Models Examining Predictors of Suicide in the Year After CSRE Administration for CSREs Within 2 Days of a Positive Columbia Suicide Severity Rating Scale Screen (Ns =81 102)
eTable 4. Clinician-Determined Risk Strata Combinations and Suicide Following CSRE Receipt (Ns = 269 198)

This supplementary material has been provided by the authors to give readers additional information about their work.

© 2025 Saulnier KG et al. *JAMA Network Open*.

eTable 1. Cox Proportional Hazards Regression Models Examining Associations With Suicide Within 30 and 365 Days of CSRE Administration Without Clinician-Determined Risk Strata (Ns = 269 372)

	Suicide within 30 days				Suicide within 365 days			
Predictor	HR	LL	UL	*p*	HR	LL	UL	*p*
Age 35-54 years (ref: 18-34 years)	1.32	0.61	2.82	0.48	0.72	0.52	1.01	0.05
Age 55+ years (ref: 18-34 years)	1.16	0.56	2.41	0.69	0.58	0.39	0.89	0.01
Female sex (ref: male)	1.16	0.51	2.65	0.73	0.70	0.44	1.11	0.13
Race: American Indian/Alaskan Native (ref: White)	0.61	0.08	4.57	0.63	1.11	0.40	3.11	0.84
Race: Asian (ref: White)	4.28	0.68	27.07	0.12	1.48	0.36	6.07	0.59
Race: Black (ref: White)	0.27	0.12	0.62	0.002	0.23	0.15	0.35	<0.001
Race: Multiracial (ref: White)	--	--	--	--	0.75	0.15	3.74	0.73
Race: Native Hawaiian/Pacific Islander (ref: White)	--	--	--	--	0.50	0.11	2.38	0.39
Race: Unknown (ref: White)	1.56	0.74	3.29	0.24	0.76	0.48	1.18	0.22
Hispanic ethnicity (ref: non-Hispanic)	0.24	0.08	0.79	0.02	0.74	0.45	1.22	0.24
Service connection (ref: no service connection)	0.32	0.19	0.54	<0.001	0.48	0.35	0.67	<0.001
Setting: ED (ref: MHC)	1.49	0.74	3.01	0.27	1.16	0.83	1.61	0.39
Setting: IP MH (ref: MHC)	1.91	0.44	8.25	0.39	1.52	0.73	3.17	0.26
Setting: IP non-MH (ref: MHC)	--	--	--	--	1.90	0.47	7.67	0.37
Setting: OP non-MH (ref: MHC)	1.13	0.74	1.74	0.57	1.09	0.86	1.38	0.47
Setting: Primary care (ref: MHC)	0.89	0.12	6.58	0.91	1.04	0.42	2.55	0.93
Setting: PCMHI (ref: MHC)	0.24	0.03	1.76	0.16	0.68	0.38	1.21	0.19
Depressive disorder	0.83	0.51	1.34	0.44	1.05	0.81	1.36	0.70
Bipolar disorder	1.09	0.62	1.91	0.77	1.24	0.90	1.71	0.19
Schizophrenia spectrum disorder	1.54	0.68	3.48	0.30	1.40	0.88	2.24	0.16
PTSD	0.87	0.57	1.32	0.50	0.76	0.58	0.99	0.04
Anxiety disorder	1.20	0.74	1.97	0.46	1.45	1.12	1.88	0.01
AUD/SUD	0.93	0.55	1.57	0.79	1.14	0.84	1.55	0.40
Insomnia disorder	1.10	0.61	1.95	0.76	1.02	0.76	1.36	0.91
CSRE Suicidal ideation	3.57	1.77	7.19	<0.001	1.91	1.40	2.61	<0.001
CSRE Firearm access	2.64	1.49	4.65	<0.001	1.56	1.14	2.14	0.006
CSRE Other lethal means access	1.43	0.91	2.25	0.12	1.02	0.82	1.28	0.85
CSRE Prior suicide attempt	0.61	0.35	1.07	0.08	1.04	0.83	1.30	0.73
CSRE Preparatory behavior	1.66	0.94	2.90	0.08	1.19	0.88	1.59	0.26
WS suicidal communication	1.35	0.89	2.06	0.16	1.20	0.96	1.50	0.10
WS direct preparations	2.24	1.32	3.78	0.003	1.60	1.11	2.31	0.01
WS seeking lethal means access	2.10	1.14	3.89	0.02	1.26	0.89	1.77	0.19
WS anger	0.51	0.30	0.86	0.01	0.57	0.45	0.72	<0.001
WS anxiety	1.83	1.17	2.88	0.01	1.16	0.94	1.44	0.17
WS guilt	1.13	0.69	1.86	0.63	1.14	0.92	1.41	0.25

© 2025 Saulnier KG et al. *JAMA Network Open*.

WS hopelessness	0.90	0.55	1.47	0.67	1.11	0.89	1.38	0.36
WS increased isolation	0.85	0.49	1.46	0.55	1.00	0.80	1.26	0.98
WS reckless behaviors	1.33	0.77	2.27	0.31	1.40	1.01	1.96	0.05
WS sleep disturbance	0.71	0.42	1.18	0.18	0.88	0.71	1.08	0.22
WS substance abuse	0.71	0.43	1.17	0.18	0.95	0.73	1.22	0.67
WS other	1.16	0.68	1.97	0.59	1.29[a]	1.03	1.61	0.03
RF history of suicidal behavior	0.99	0.62	1.58	0.96	1.16	0.89	1.50	0.28
RF recent stressor	1.17	0.77	1.79	0.46	0.98	0.78	1.23	0.88
RF access to lethal means	0.66	0.40	1.09	0.10	0.97	0.75	1.24	0.78
RF history of psychiatric hospitalization	1.69	1.06	2.69	0.03	1.73	1.36	2.22	<0.001
RF psychiatric conditions	0.84	0.53	1.31	0.44	1.01	0.82	1.25	0.91
RF history of nonsuicidal self-injury	0.97	0.53	1.78	0.91	1.07	0.77	1.49	0.68
RF recent losses	0.99	0.63	1.55	0.97	0.98	0.78	1.24	0.89
RF pre-existing risk factors (e.g., trauma)	0.67	0.42	1.06	0.09	0.78[a]	0.63	0.98	0.03
RF chronic medical condition	1.20	0.75	1.91	0.44	1.18	0.95	1.47	0.14
RF marginalized group status	0.35	0.08	1.47	0.15	1.04	0.55	1.97	0.91
RF recent transition from military	0.47	0.11	2.08	0.32	0.40	0.23	0.72	0.002
RF other	0.91	0.41	2.02	0.82	1.25	0.87	1.79	0.22
PF access to healthcare	0.95	0.58	1.54	0.83	1.05	0.86	1.28	0.67
PF motivation for medical treatment	0.85	0.55	1.31	0.46	1.08	0.91	1.30	0.38
PF access to MH care	0.96	0.62	1.48	0.84	0.97	0.80	1.16	0.71
PF motivation for MH treatment	0.73	0.51	1.04	0.08	1.01	0.83	1.22	0.96
PF positive interpersonal relationships	1.50	0.96	2.32	0.07	1.13	0.93	1.37	0.22
PF significant other	0.87	0.50	1.52	0.63	1.14	0.87	1.51	0.34
PF caregiving responsibilities	1.08	0.59	1.97	0.81	0.87	0.65	1.16	0.34
PF hope for the future	1.13	0.74	1.72	0.57	1.04	0.87	1.25	0.67
PF personal traits/beliefs against suicide	1.00	0.65	1.54	1.00	0.79[a]	0.65	0.96	0.02
PF religious beliefs against suicide	0.81	0.48	1.36	0.42	0.90	0.70	1.16	0.42
PF cultural connections	1.01	0.57	1.78	0.97	0.83	0.60	1.15	0.27
PF social support	0.93	0.63	1.37	0.72	0.94	0.76	1.16	0.55
PF desire to live	1.14	0.71	1.83	0.59	0.96	0.78	1.18	0.71
PF other	1.13	0.57	2.25	0.72	1.21	0.84	1.75	0.31

Note. CSRE = Comprehensive Suicide Risk Evaluation. ED = Emergency department. IP = Inpatient. MHC = Mental Health Clinic. MH = Mental health. OP = Outpatient. PCMHI = Primary care-mental health integration. PTSD = Posttraumatic stress disorder. AUD/SUD = Alcohol use disorder/substance use disorder. WS = Warning sign. RF = Risk factor. PF = Protective factor.

[a] Effect did not meet criteria for being “statistically and clinically significant” (i.e., hazard ratios [HRs]≤0.77 or ≥1.30, $p \leq 0.05$.

© 2025 Saulnier KG et al. *JAMA Network Open*.

eTable 2. Cox Proportional Hazards Regression Models Examining Predictors of Suicide in the Year After CSRE Administrations for Initial CSREs (Ns = 153 648)

	Suicide within 30 days				Suicide within 365 days			
Predictor	HR	LL	UL	*p*	HR	LL	UL	*p*
Age 35-54 years (ref: 18-34 years)	1.65	0.72	3.77	0.24	0.81	0.61	1.07	0.13
Age 55+ years (ref: 18-34 years)	1.43	0.61	3.35	0.41	0.56	0.41	0.77	<0.001
Female sex (ref: male)	0.52	0.19	1.47	0.22	0.67	0.46	0.98	0.04
Race: American Indian/Alaskan Native (ref: White)	--	--	--	--	1.09	0.45	2.65	0.86
Race: Asian (ref: White)	--	--	--	--	0.62	0.20	1.94	0.41
Race: Black (ref: White)	0.32	0.13	0.81	0.02	0.31	0.21	0.45	<0.001
Race: Multiracial (ref: White)	--	--	--	--	0.49	0.16	1.54	0.22
Race: Native Hawaiian/Pacific Islander (ref: White)	--	--	--	--	0.26	0.04	1.84	0.18
Race: Unknown (ref: White)	1.59	0.77	3.28	0.21	0.80	0.53	1.19	0.27
Hispanic ethnicity (ref: non-Hispanic)	0.38	0.09	1.58	0.18	0.63	0.40	1.00	0.05
Service connection (ref: no service connection)	0.30	0.17	0.52	<0.001	0.42	0.34	0.53	<0.001
Setting: ED (ref: MHC)	1.15	0.47	2.84	0.76	1.12	0.73	1.72	0.59
Setting: IP MH (ref: MHC)	--	--	--	--	1.02	0.25	4.13	0.98
Setting: IP non-MH (ref: MHC)	--	--	--	--	2.86	0.40	20.44	0.30
Setting: OP non-MH (ref: MHC)	1.00	0.51	1.96	1.00	1.21	0.91	1.61	0.18
Setting: Primary care (ref: MHC)	1.06	0.14	7.87	0.95	1.17	0.43	3.16	0.76
Setting: PCMHI (ref: MHC)	0.26	0.04	1.88	0.18	0.57	0.30	1.08	0.08
Depressive disorder	0.72	0.42	1.24	0.23	0.93	0.73	1.18	0.53
Bipolar disorder	0.93	0.44	1.98	0.86	1.00	0.74	1.35	1.00
Schizophrenia spectrum disorder	1.51	0.65	3.52	0.34	1.41	0.98	2.02	0.06
PTSD	0.81	0.45	1.46	0.48	0.73	0.57	0.93	0.01
Anxiety disorder	0.95	0.54	1.66	0.86	1.30	1.03	1.63	0.03
AUD/SUD	1.00	0.57	1.78	0.99	1.17	0.92	1.50	0.20
Insomnia disorder	1.35	0.72	2.51	0.35	1.10	0.84	1.44	0.48
CSRE Suicidal ideation	4.22	1.79	9.94	0.001	1.81	1.29	2.54	<0.001
CSRE Firearm access	3.23	1.65	6.33	<0.001	2.06	1.50	2.84	<0.001
CSRE Other lethal means access	1.33	0.70	2.54	0.38	1.10	0.82	1.47	0.54
CSRE Prior suicide attempt	0.77	0.37	1.60	0.48	1.07	0.80	1.43	0.63
CSRE Preparatory behavior	1.38	0.61	3.11	0.43	0.99	0.66	1.48	0.96
WS suicidal communication	1.83	1.00	3.36	0.05	1.32	1.00	1.74	0.05
WS direct preparations	1.91	0.83	4.40	0.13	1.24	0.82	1.88	0.32
WS seeking lethal means access	2.44	1.09	5.49	0.03	1.31	0.87	1.99	0.20
WS anger	0.42	0.19	0.93	0.03	0.61	0.45	0.84	0.002

© 2025 Saulnier KG et al. *JAMA Network Open*.

WS anxiety	1.79	0.99	3.23	0.05	1.14	0.88	1.49	0.33
WS guilt	1.41	0.74	2.70	0.30	1.24	0.93	1.66	0.14
WS hopelessness	1.09	0.58	2.02	0.80	1.16	0.88	1.54	0.30
WS increased isolation	0.62	0.31	1.24	0.17	0.79	0.59	1.06	0.12
WS reckless behaviors	0.60	0.18	2.05	0.42	1.17	0.78	1.75	0.44
WS sleep disturbance	0.77	0.41	1.42	0.40	0.84	0.64	1.11	0.22
WS substance abuse	0.70	0.33	1.50	0.36	0.93	0.69	1.26	0.64
WS other	1.39	0.69	2.79	0.35	1.33	0.97	1.82	0.008
RF history of suicidal behavior	1.13	0.58	2.19	0.73	1.32	0.99	1.75	0.06
RF recent stressor	1.13	0.67	1.93	0.64	1.00	0.79	1.26	0.99
RF access to lethal means	0.78	0.39	1.55	0.48	0.73	0.53	1.01	0.05
RF history of psychiatric hospitalization	1.56	0.88	2.78	0.13	1.31	1.02	1.67	0.03
RF psychiatric conditions	0.66	0.37	1.15	0.14	1.02	0.78	1.33	0.91
RF history of nonsuicidal self-injury	0.26	0.04	1.92	0.19	0.67	0.42	1.07	0.09
RF recent losses	0.92	0.50	1.70	0.78	1.07	0.83	1.39	0.60
RF pre-existing risk factors (e.g., trauma)	0.93	0.50	1.73	0.83	0.94	0.73	1.21	0.63
RF chronic medical condition	0.81	0.46	1.41	0.45	0.97	0.76	1.23	0.78
RF marginalized group status	--	--	--	--	1.07	0.61	1.89	0.81
RF recent transition from military	0.50	0.07	3.78	0.50	0.53	0.27	1.05	0.07
RF other	0.62	0.19	2.02	0.43	1.23	0.82	1.83	0.31
PF access to healthcare	0.90	0.51	1.58	0.72	1.03	0.80	1.32	0.84
PF motivation for medical treatment	0.73	0.41	1.29	0.28	1.25	0.98	1.59	0.07
PF access to MH care	0.93	0.53	1.63	0.81	1.07	0.83	1.37	0.61
PF motivation for MH treatment	0.66	0.38	1.14	0.13	0.93	0.72	1.19	0.54
PF positive interpersonal relationships	1.55	0.88	2.72	0.13	1.11	0.88	1.40	0.39
PF significant other	0.97	0.55	1.71	0.91	0.96	0.75	1.23	0.74
PF caregiving responsibilities	0.70	0.36	1.37	0.30	0.81	0.62	1.07	0.14
PF hope for the future	0.92	0.51	1.66	0.77	0.99	0.77	1.27	0.92
PF personal traits/beliefs against suicide	1.03	0.58	1.82	0.92	0.80	0.63	1.03	0.08
PF religious beliefs against suicide	1.07	0.58	1.97	0.83	0.99	0.75	1.30	0.93
PF cultural connections	0.70	0.21	2.34	0.56	0.80	0.49	1.32	0.38
PF social support	1.08	0.61	1.93	0.79	0.94	0.73	1.21	0.62
PF desire to live	1.34	0.73	2.47	0.34	0.96	0.74	1.25	0.77
PF other	0.80	0.25	2.60	0.71	0.99	0.63	1.56	0.98
High acute suicide risk (ref: low)	1.19	0.46	3.08	0.72	1.39	0.92	2.11	0.12
Intermediate acute suicide risk (ref: low)	1.28	0.65	2.53	0.47	1.50	1.13	2.00	0.006
High chronic suicide risk (ref: low)	1.09	0.42	2.84	0.86	1.50	0.98	2.29	0.06
Intermediate chronic suicide risk (ref: low)	0.94	0.49	1.81	0.86	1.25	0.92	1.69	0.15

© 2025 Saulnier KG et al. *JAMA Network Open*.

Note. CSRE = Comprehensive Suicide Risk Evaluation. ED = Emergency department. IP = Inpatient. MHC = Mental Health Clinic. MH = Mental health. OP = Outpatient. PCMHI = Primary care-mental health integration. PTSD = Posttraumatic stress disorder. AUD/SUD = Alcohol use disorder/substance use disorder. WS = Warning sign. RF = Risk factor. PF = Protective factor.

© 2025 Saulnier KG et al. *JAMA Network Open*.

eTable 3. Cox Proportional Hazards Regression Models Examining Predictors of Suicide in the Year After CSRE Administration for CSREs Within 2 Days of a Positive Columbia Suicide Severity Rating Scale Screen (Ns =81 102)

	Suicide within 30 days				Suicide within 365 days			
Predictor	HR	LL	UL	*p*	HR	LL	UL	*p*
Age 35-54 years (ref: 18-34 years)	1.63	0.64	4.18	0.31	0.77	0.51	1.17	0.22
Age 55+ years (ref: 18-34 years)	1.36	0.55	3.37	0.50	0.69	0.43	1.12	0.13
Female sex (ref: male)	1.57	0.56	4.38	0.39	0.87	0.49	1.53	0.62
Race: American Indian/Alaskan Native (ref: White)	--	--	--	--	0.60	0.14	2.64	0.50
Race: Asian (ref: White)	3.30	0.48	22.67	0.23	1.10	0.22	5.57	0.91
Race: Black (ref: White)	0.23	0.07	0.80	0.02	0.25	0.14	0.44	<0.001
Race: Multiracial (ref: White)	--	--	--	--	0.63	0.13	3.00	0.56
Race: Native Hawaiian/Pacific Islander (ref: White)	--	--	--	--	0.77	0.11	5.46	0.79
Race: Unknown (ref: White)	1.06	0.39	2.86	0.92	0.65	0.35	1.22	0.18
Hispanic ethnicity (ref: non-Hispanic)	0.16	0.02	1.20	0.08	0.58	0.29	1.14	0.11
Service connection (ref: no service connection)	0.43	0.24	0.78	0.01	0.52	0.36	0.75	<0.001
Setting: ED (ref: MHC)	1.02	0.44	2.40	0.964	1.04	0.69	1.56	0.85
Setting: IP MH (ref: MHC)	2.13	0.45	10.07	0.34	1.47	0.66	3.26	0.35
Setting: IP non-MH (ref: MHC)	--	--	--	--	2.35	0.32	17.28	0.40
Setting: OP non-MH (ref: MHC)	0.96	0.50	1.84	0.91	0.83	0.59	1.18	0.30
Setting: Primary care (ref: MHC)	0.97	0.13	7.53	0.98	1.17	0.47	2.91	0.74
Setting: PCMHI (ref: MHC)	--	--	--	--	0.42	0.19	0.91	0.03
Depressive disorder	0.64	0.37	1.11	0.11	0.97	0.72	1.31	0.85
Bipolar disorder	1.18	0.58	2.37	0.65	1.37	0.96	1.91	0.09
Schizophrenia spectrum disorder	1.16	0.47	2.81	0.75	1.39	0.83	2.32	0.21
PTSD	0.83	0.50	1.38	0.48	0.67	0.49	0.91	0.01
Anxiety disorder	1.02	0.53	1.94	0.96	1.40	1.02	1.93	0.04
AUD/SUD	1.07	0.57	2.03	0.83	1.18	0.83	1.69	0.35
Insomnia disorder	1.15	0.52	2.57	0.73	1.03	0.71	1.50	0.89
CSRE Suicidal ideation	1.48	0.37	5.99	0.58	1.02	0.62	1.67	0.94
CSRE Firearm access	3.14	1.55	6.34	0.002	1.38	0.98	1.97	0.07
CSRE Other lethal means access	1.46	0.77	2.78	0.25	1.00	0.75	1.35	0.98
CSRE Prior suicide attempt	0.79	0.41	1.52	0.48	0.92	0.69	1.22	0.55
CSRE Preparatory behavior	1.18	0.53	2.66	0.69	1.06	0.73	1.54	0.77
WS suicidal communication	0.95	0.58	1.57	0.85	1.00	0.77	1.31	0.99
WS direct preparations	1.81	0.88	3.73	0.11	1.32	0.88	2.00	0.18
WS seeking lethal means access	2.15	1.03	4.50	0.04	1.29	0.89	1.87	0.18
WS anger	0.46	0.24	0.89	0.02	0.56	0.42	0.76	<0.001

© 2025 Saulnier KG et al. *JAMA Network Open*.

WS anxiety	1.194	1.07	3.22	0.03	1.20	0.92	1.56	0.18
WS guilt	1.23	0.73	2.08	0.44	1.22	0.94	1.57	0.13
WS hopelessness	0.86	0.46	1.60	0.63	0.98	0.76	1.26	0.86
WS increased isolation	0.71	0.36	1.42	0.33	1.05	0.79	1.39	0.76
WS reckless behaviors	0.87	0.40	1.88	0.72	1.20	0.83	1.74	0.33
WS sleep disturbance	0.81	0.43	1.52	0.51	0.81	0.63	1.06	0.13
WS substance abuse	0.91	0.52	1.60	0.74	0.98	0.72	1.33	0.89
WS other	0.62	0.27	1.40	0.24	1.16	0.85	1.58	0.34
RF history of suicidal behavior	0.79	0.41	1.42	0.43	0.96	0.71	1.30	0.78
RF recent stressor	1.22	0.68	2.17	0.51	0.88	0.68	1.15	0.34
RF access to lethal means	0.67	0.33	1.33	0.25	1.02	0.73	1.43	0.92
RF history of psychiatric hospitalization	1.43	0.80	2.55	0.23	1.28	0.96	1.71	0.10
RF psychiatric conditions	0.92	0.47	1.77	0.79	0.92	0.69	1.22	0.56
RF history of nonsuicidal self-injury	0.77	0.31	1.87	0.56	0.78	0.49	1.26	0.31
RF recent losses	1.02	0.57	1.82	0.95	0.94	0.68	1.29	0.69
RF pre-existing risk factors (e.g., trauma)	0.71	0.35	1.44	0.34	0.89	0.64	1.22	0.46
RF chronic medical condition	1.06	0.56	2.01	0.85	1.05	0.78	1.41	0.77
RF marginalized group status	0.38	0.05	2.89	0.35	1.01	0.48	2.13	0.97
RF recent transition from military	--	--	--	--	0.21	0.07	0.65	0.01
RF other	1.07	0.44	2.64	0.88	1.33	0.91	1.93	0.14
PF access to healthcare	1.00	0.49	2.01	0.99	1.19	0.90	1.58	0.22
PF motivation for medical treatment	0.78	0.41	1.48	0.45	1.00	0.78	1.29	0.98
PF access to MH care	0.96	0.52	1.77	0.90	0.96	0.74	1.25	0.75
PF motivation for MH treatment	0.76	0.48	1.22	0.26	0.90	0.70	1.16	0.41
PF positive interpersonal relationships	1.20	0.61	2.36	0.61	1.13	0.86	1.49	0.39
PF significant other	0.46	0.22	0.96	0.04	1.01	0.71	1.44	0.96
PF caregiving responsibilities	1.44	0.66	3.14	0.37	0.94	0.66	1.35	0.73
PF hope for the future	1.25	0.69	2.29	0.46	1.23	0.96	1.58	0.10
PF personal traits/beliefs against suicide	1.05	0.54	2.04	0.88	0.89	0.69	1.16	0.40
PF religious beliefs against suicide	1.08	0.55	2.11	0.83	0.94	0.71	1.25	0.66
PF cultural connections	1.44	0.67	3.12	0.35	0.86	0.53	1.40	0.54
PF social support	0.86	0.50	1.47	0.58	0.93	0.71	1.22	0.59
PF desire to live	0.86	0.42	1.73	0.66	1.09	0.82	1.45	0.57
PF other	1.23	0.50	2.99	0.65	1.42	0.94	2.15	0.09
High acute suicide risk (ref: low)	1.18	0.51	2.68	0.70	1.20	0.80	1.81	0.38
Intermediate acute suicide risk (ref: low)	1.05	0.58	1.88	0.88	1.15	0.86	1.52	0.35
High chronic suicide risk (ref: low)	1.42	0.56	3.58	0.46	1.63	1.06	2.50	0.03
Intermediate chronic suicide risk (ref: low)	0.87	0.42	1.83	0.71	1.13	0.80	1.60	0.49

© 2025 Saulnier KG et al. *JAMA Network Open*.

Note. CSRE = Comprehensive Suicide Risk Evaluation. ED = Emergency department. IP = Inpatient. MHC = Mental Health Clinic. MH = Mental health. OP = Outpatient. PCMHI = Primary care-mental health integration. PTSD = Posttraumatic stress disorder. AUD/SUD = Alcohol use disorder/substance use disorder. WS = Warning sign. RF = Risk factor. PF = Protective factor.

© 2025 Saulnier KG et al. *JAMA Network Open*.

eTable 4. Clinician-Determined Risk Strata Combinations and Suicide Following CSRE Receipt (Ns = 269 198)

	Suicide within 30 days				Suicide within 365 days			
Predictor	HR	LL	UL	*p*	HR	LL	UL	*p*
Acute by chronic suicide risk				<0.001				<0.001
Low acute, low chronic	ref				ref			
Low acute, intermediate chronic	2.02	1.16	3.52	0.01	2.19	1.65	2.91	<0.001
Low acute, high chronic	4.37	1.88	10.18	<0.001	3.38	2.18	5.23	<0.001
Intermediate acute, low chronic	3.04	1.24	7.47	0.02	2.64	1.78	3.92	<0.001
Intermediate acute, intermediate chronic	2.38	1.37	4.15	0.002	2.78	2.16	3.58	<0.001
Intermediate acute, high chronic	4.07	1.95	8.48	<0.001	4.48	3.06	6.57	<0.001
High acute, low chronic[a]	--	--	--	--	--	--	--	--
High acute, intermediate chronic	4.66	2.36	9.22	<0.001	3.55	2.39	5.29	<0.001
High acute, high chronic	4.68	2.28	9.58	<0.001	4.69	3.34	6.60	<0.001

Note. HR = Hazard ratio. LL = Lower limit. UL = Upper limit.
[a]Cell sizes < 10 are suppressed per privacy guidelines.

© 2025 Saulnier KG et al. *JAMA Network Open*.
