# Supplementary material for: Suicide Risk Evaluations and Suicide in the Veterans Health Administration
Source: JAMA Netw Open. 2025 Feb 25;8(2):e2461559. doi: 10.1001/jamanetworkopen.2024.61559 (PMC11862973; doi:10.1001/jamanetworkopen.2024.61559)
Supplement: Supplement 2. — Data Sharing Statement [file jamanetwopen-e2461559-s002.pdf]

Data Sharing Statement

Saulnier. Suicide Risk Evaluations and Suicide in the Veterans Health Administration. *JAMA Netw Open*. Published February 25, 2025. doi:10.1001/jamanetworkopen.2024.61559

Data
Data available: No
